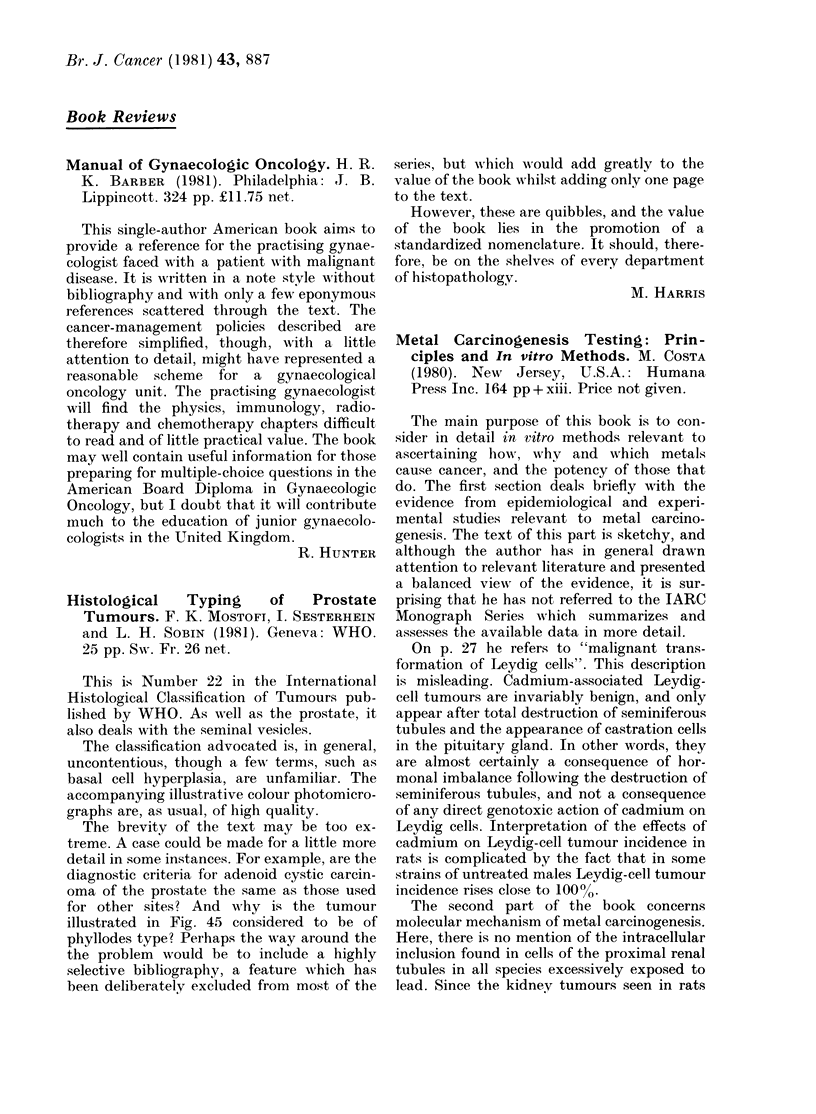# Manual of Gynaecologic Oncology

**Published:** 1981-06

**Authors:** R. Hunter


*Br. J. Cancer* (1981) **43**, 887

### *Book Reviews*

**Manual of Gynaecologic Oncology.** H. R. K. Barber (1981). Philadelphia: J. B. Lippincott. 324 pp. £11.75 net.

This single-author American book aims to provide a reference for the practising gynaecologist faced with a patient with malignant disease. It is written in a note style without bibliography and with only a few eponymous references scattered through the text. The cancer-management policies described are therefore simplified, though, with a little attention to detail, might have represented a reasonable scheme for a gynaecological oncology unit. The practising gynaecologist will find the physics, immunology, radiotherapy and chemotherapy chapters difficult to read and of little practical value. The book may well contain useful information for those preparing for multiple-choice questions in the American Board Diploma in Gynaecologic Oncology, but I doubt that it will contribute much to the education of junior gynaecolo-cologists in the United Kingdom.

R. Hunter